# The New p.F1700L LRRK2 Variant Causes Parkinson's Disease by Extensively Increasing Kinase Activity

**DOI:** 10.1002/mds.29385

**Published:** 2023-03-27

**Authors:** Max Borsche, Neringa Pratuseviciute, Susen Schaake, Frauke Hinrichs, Gabriel Morel, Jan Uter, Katja Lohmann, Christine Klein, Dario R. Alessi, Johann Hagenah, Esther Sammler

**Affiliations:** ^1^ Institute of Neurogenetics University of Lübeck Lübeck Germany; ^2^ Department of Neurology University of Lübeck Lübeck Germany; ^3^ MRC Protein Phosphorylation and Ubiquitylation Unit, School of Life Sciences University of Dundee Dundee United Kingdom; ^4^ Department of Neurology, Westküstenklinikum Heide Heide Germany; ^5^ Molecular and Clinical Medicine, Ninewells Hospital and Medical School University of Dundee Dundee United Kingdom

Variants in *LRRK2* represent the most frequent cause of clinically classical monogenic Parkinson's disease (PD).^1^ Altered LRRK2 protein function boosts neuroinflammation, impairs vesicle trafficking, and affects ciliogenesis within the striatum.^2^ Because a relevant fraction of the more than 1000 identified *LRRK2* variants^3^ is not pathogenic, determining pathogenicity for single variants is crucial, particularly because LRRK2 kinase inhibitors have entered phase 3 trials.^4^ Notably, we have already established an analytic workflow to determine kinase activity and decipher the pathogenicity of single *LRRK2* variants in vitro^5^ and in vivo.^6^

In this letter, we report on a 74‐year‐old male patient from northern Germany with advanced typical PD (Unified Parkinson's Disease Rating Scale Part III: 33/108 points, Hoehn and Yahr stage 3–4) without relevant tremor. The age at onset was 67 years. The disease course was slowly progressive, he experienced a good response to dopaminergic therapy, and dementia was absent. Family history was suggestive of autosomal dominant inheritance, with the father and two brothers also diagnosed with PD. However, the father was already deceased, one brother was not available for examination, and the second brother died early after giving blood for genetic investigation. Both brothers carried a new p.F1700L (NM_198578.4: c.5098T>C) variant in *LRRK2*, initially detected by gene panel analysis in the deceased brother and further investigated by Sanger sequencing. The variant is rated as a variant of uncertain significance according to the criteria of the American College of Medical Genetics (assessed by Franklin: https://franklin.genoox.com/) and is not listed in gnomAD (https://gnomad.broadinstitute.org/). In silico prediction suggested pathogenicity based on a Combined Annotation Dependent Depletion (CADD) score (https://cadd.gs.washington.edu/) of 27.3. The variant is located within the C terminus of the Ras of complex protein B scaffolding domain.^5^

We investigated LRRK2 kinase pathway activity in a heterologous transient overexpression system in HEK293 cells as described previously^5^ (Fig. 1A). We then analyzed LRRK2 activity in vivo in fresh peripheral blood, simultaneously collected from a p.F1700L carrier and a sex‐matched healthy control subject (Fig. 1B). The resulting LRRK2‐dependent pRab10^Thr73^ phosphorylation level mirrors LRRK2 kinase activation status.^6^ Both experiments demonstrated significant LRRK2 kinase hyperactivation because of the p.F1700L variant. Notably, p.F1700L demonstrated LRRK2 kinase hyperactivation similar to the neighboring p.Y1699C variant, which has the highest degree of kinase activity among all *LRRK2* variants investigated thus far in the HEK293 assay (Fig. 1A). Moreover, we found a 7‐ to 8‐fold increase in Rab10 phosphorylation levels in vivo (Fig. 1B).

**FIG 1:**
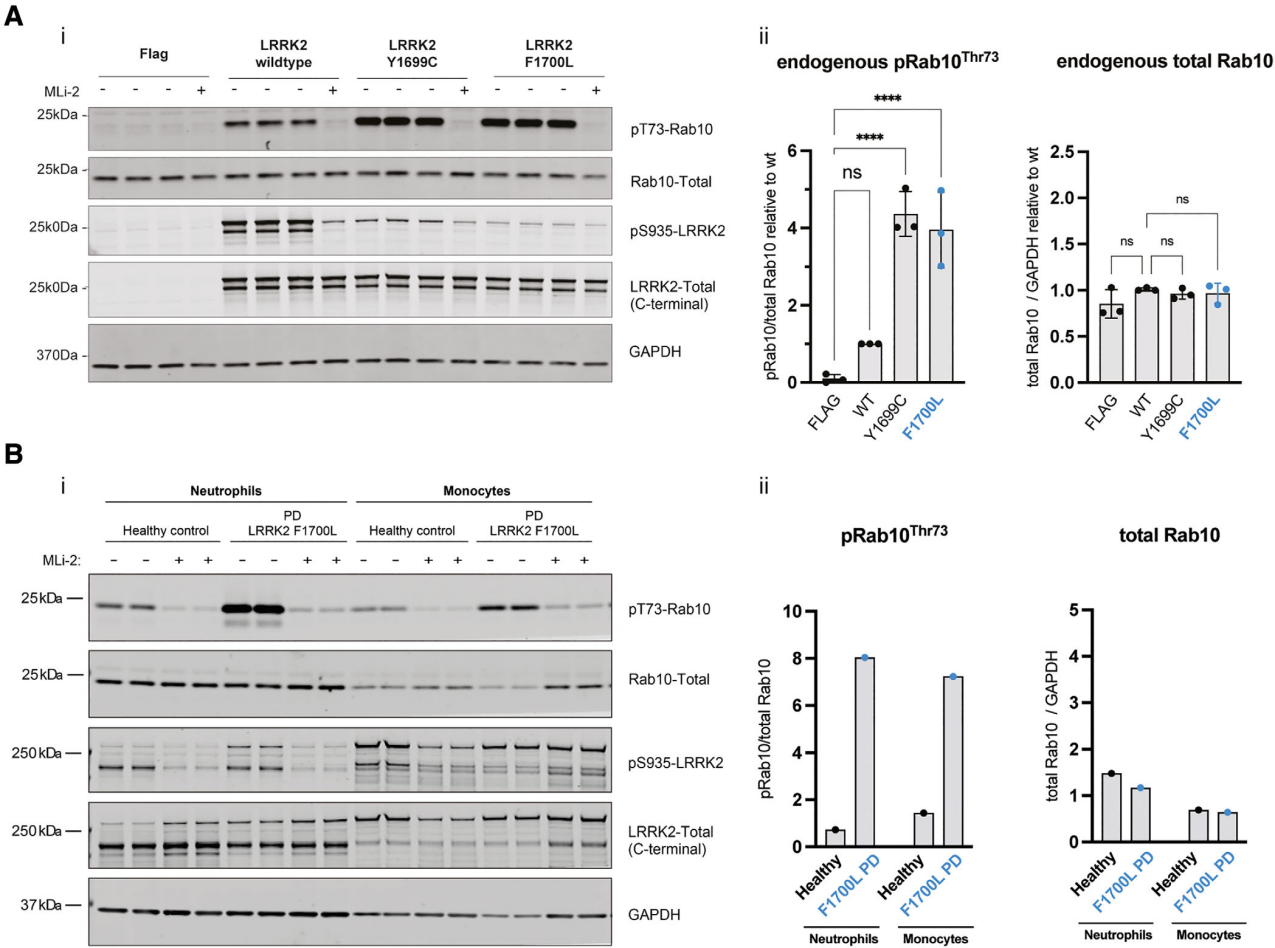
LRRK2 kinase hyperactivity in vitro and in vivo as a result of the newly identified p.F1700L pathogenic variant. (**A**) In vitro characterization of the novel *LRRK2* p.F1700L variant in comparison with the neighboring pathogenic *LRRK2* p.Y1699C variant in an established HEK293 overexpression system, followed by *LI‐COR* Odyssey immunoblotting (**i**) and quantification of LRRK2 kinase activity relative to LRRK2 wild type (wt). LRRK2‐dependent phosphorylation of endogenous Rab10 at threonine 73 (pRab10^Thr73^) was used as a readout for LRRK2 kinase activity, and the LRRK2‐specific small molecule inhibitor MLi‐2 at 200 nM for 90 minutes to demonstrate LRRK2 kinase dependency of pRab10^Thr73^ as before.^5^ The p.F1700L variant demonstrated significant LRRK2 kinase activity with a 4‐fold increase of LRRK2‐dependent Rab10 phosphorylation compared with LRRK2 wild type similar in effect size to *LRRK2* p.Y1699C. Endogenous Rab10 levels did not differ (**ii**). Each data point represents a biological replicate experiment. Data were analyzed using one‐way ANOVA with multiple comparisons test. Statistical significance was determined from three replicate values for each variant and represented with *P* values (****P < 0.0001). (**B**) In vivo analysis of kinase activity in patient‐derived neutrophils and monocytes. Fresh blood was taken from the Parkinson's disease patient carrying the *LRRK2* p.F1700L variant and a sex‐matched healthy control subject. Immunomagnetic negative isolation of peripheral blood neutrophils and monocytes was performed, and each sample was then split into two batches for treatment with and without the specific LRRK2 kinase inhibitor MLi‐2 before cell lysis as before.^6^, ^7^ As with the HEK293 overexpression assay, LRRK2‐dependent Rab10 phosphorylation (pRab10^Thr73^) as a readout for LRRK2 kinase activity was significantly increased in both peripheral blood neutrophils and monocytes derived from the *LRRK2* p.F1700L variant carrier compared with the control. [Color figure can be viewed at wileyonlinelibrary.com]

Together, we provide robust evidence for the pathogenicity of the newly identified p.F1700L variant in *LRRK2*, demonstrating that this substitution is among the variants with the highest kinase activity of all *LRRK2* variants investigated to date and impacts protein function more profoundly than, for example, the frequent p.G2019S variant.^5^, ^6^ Thus, carriers of a p.F1700L variant should be included in clinical trials targeting LRRK2 kinase activity already initiated or soon commencing.^4^ Finally, we confirmed the applicability and usefulness of the applied assays to determine the pathogenicity of *LRRK2* variants of uncertain significance. Further studies should focus on an association between the degree of kinase activity and penetrance, disease onset, and disease severity.

## Ethics Statement

This study was approved by the ethics committee of the University of Lübeck and performed according to the Declaration of Helsinki.

## Author Roles

(1) Research project: A. Conception, B. Organization, C. Execution; (2) Statistical Analysis: A. Design, B. Execution, C. Review and Critique; (3) Manuscript: A. Writing of the first draft, B. Review and Critique.

M.B.: 1A, 1B, 1C, 2C, 3A, 3B.

N.P.: 1B, 1C, 2A, 2B, 3B.

S.S.: 1B, 1C, 3B.

F.H.: 1B, 1C, 3B.

G.M.: 1B, 1C, 3B.

J.U.: 1B, 1C, 3B.

K.L.: 1A, 1B, 1C, 3B.

C.K.: 1A, 1B, 1C, 3B.

D.R.A.: 1A, 1B, 1C, 3B.

J.H.: 1A, 1B, 1C, 3B.

E.S.: 1A, 1B, 1C, 2A, 2B, 3A, 3B.

## Full Financial Disclosures for the Previous 12 Months

Max Borsche: Employment, University of Lübeck and University Hospital Schleswig‐Holstein, Campus Lübeck; Honoraria, Ipsen Pharma; Grants, German Research Foundation (DFG)‐funded Clinician Scientist School, University of Lübeck. Neringa Pratuseviciute: Employment, Carnegie Trust–funded PhD student, University of Dundee. Susen Schaake: Employment, University of Lübeck and University Hospital Schleswig‐Holstein, Campus Lübeck. Frauke Hinrichs: Employment, University of Lübeck and University Hospital Schleswig‐Holstein, Campus Lübeck. Gabriel Morel: Employment, Undergraduate placement student at University of Dundee. Jan Uter: Employment, University of Lübeck and University Hospital Schleswig‐Holstein, Campus Lübeck. Katja Lohmann: Employment, University of Lübeck; Grants, German Research Foundation (DFG), Parkinson's foundation, Damp Foundation, The Michael J. Fox Foundation (MJFF; GP2 project). Christine Klein: Consultancies, Centogene, Retromer Therapeutics; Employment, University of Lübeck and University Hospital Schleswig‐Holstein, Campus Lübeck; Honoraria, Desitin and Bial; Royalties, Oxford University Press; Grants, DFG, MJFF, Aligning Science Across Parkinson's. Dario R. Alessi: Employment, University of Dundee; Grants, Medical Research Council UK. Johann Hagenah: Employment, Westküstenklinikum Heide. Esther Sammler: Employment, University of Dundee, UK; Honoraria, Bial, MJFF; Grants, MJFF, Chief Scientist Office, Scotland, UK (personal fellowship).

## Data Availability

The data presented in this study can be received from the corresponding author upon reasonable request.
